# Parental knowledge and experience of kangaroo care for preterm infants in neonatal intensive care unit: a qualitative study

**DOI:** 10.3389/fpubh.2026.1704674

**Published:** 2026-02-25

**Authors:** Yasmine Alabbasi, Alya Elgamri, Yara Abdullah Al-Joher, Wajd Salem Al-Amri, Alanoud Daher Al-Sadoun, Shouq Mansour Kaabi, Sadeem Mtaab Al-Mutairi, Renad Ali Al-Anizi, Wejdan Ahmed Al-Haqwi, Fayzah Alhussain

**Affiliations:** 1 Department of Maternity and Pediatric Nursing, College of Nursing, Princess Nourah bint Abdulrahman University, Riyadh, Saudi Arabia; 2 Department of Orthodontics, Pediatric Dentistry, and Preventive Dentistry, Faculty of Dentistry, University of Khartoum, Khartoum, Sudan; 3 College of Nursing, Princess Nourah Bint Abudlrahman University, Riyadh, Saudi Arabia

**Keywords:** experience, kangaroo care, NICU, parents, premature infants

## Abstract

**Background:**

Kangaroo care is a low-cost, evidence-based neonatal intervention known to reduce morbidity and promote infant development and parental bonding. However, in NICUs in Saudi Arabia, parent uptake remains low, and little is known about how cultural, educational, and institutional factors shape parents’ experience and decision-making.

**Objective:**

This study aimed to assess parental knowledge and experiences regarding kangaroo care in Saudi Arabia and explore the factors influencing their decisions to practice it.

**Methods:**

A phenomenological qualitative research design was employed. A purposive sampling was used to select parents of stable preterm infants (32–37 weeks’ gestational age), in the NICU using semi-structured interviews. Data was analyzed using reflexive thematic analysis.

**Results:**

Eighteen mothers (aged 28–40 years) were interviewed. From the analysis, seven themes and five sub-themes were constructed, emphasizing the profound emotional, physical, and relational impact of kangaroo care on mothers. Mothers described kangaroo care as empowering, supporting infant recovery, enhancing breastfeeding, and fulfilling psychological and spiritual needs, often drawing strength from religious practices. A significant concern was the timing of kangaroo care education, which was typically provided after delivery when mothers felt overwhelmed, leading participants to advocate for antenatal education to improve awareness and preparedness. Additionally, multiple mothers attributed their husbands’ limited participation to hesitancy and fear.

**Conclusion:**

The study emphasizes the significance of early, culturally sensitive education and the routine incorporation of kangaroo care into neonatal care protocols, as endorsed by clinical guidelines and institutional policies. The findings underscore the central role of cultural and religious values in shaping parental experiences and emphasize the need for healthcare systems to adopt holistic, family-centered approaches. Implementing early education and culturally tailored support can enhance parental engagement, maternal empowerment, and neonatal outcomes, ultimately improving care during this critical period. This approach not only benefits individual families but also contributes to the advancement of public health and pediatric care in Saudi Arabia and beyond.

## 1 Introduction

According to the World Health Organization (WHO), an estimated 13.4 million infants were born preterm in 2020, representing nearly one in 10 live births worldwide (1). Infants born preterm exhibit widespread structural and functional immaturity across multiple organ systems, making the transition from the protected intrauterine to the extrauterine environment a substantial physiological challenge. The abrupt and pronounced shifts in temperature, pressure, and oxygenation after birth can significantly disrupt respiratory adaptation in these preterm infants (2, 3). In November 2022, the WHO issued a major revision to its global guidelines on the care of preterm and low-birth-weight infants. From that point onward, kangaroo mother care (KMC)—defined as skin-to-skin contact with a parent or caregiver, coupled with exclusive breastfeeding – became the recommended default care model for all preterm infants. WHO explicitly instructed that such care should begin as early as immediately after birth, be provided for as many hours per day as possible in hospitals (4). This change reflects strong, high-certainty evidence showing that routine early kangaroo care significantly increases survival, supports thermoregulation, reduces infection and hypothermia risks, and promotes optimal growth and neurodevelopment (4).

Kangaroo care provides a variety of important benefits for not only preterm infants, but also their parents. A recent study involving mothers who practiced KMC three to four times daily following emergency cesarean delivery found that serum oxytocin levels rose substantially, while cortisol levels decreased and prolactin surged, which facilitated milk production. These changes were accompanied by reduced postpartum inflammation and earlier initiation of breastfeeding, suggesting a compounded benefit from neuroendocrine activation (5). In a Chinese Neonatal Intensive Care Unit (NICU) trial, mothers who followed a four-week skin-to-skin regimen reported significantly lower psychological distress across Symptom Checklist-90 subscales (SCL-90), such as anxiety, depression, and hostility, and exhibited measurable improvements in sleep quality through the Athens Insomnia Scale, compared to standard care (6). Conversely, a phenomenological study in an Ethiopian NICU found that mothers of preterm infants experienced both distressing and reassuring emotions. Their experiences were negatively affected by medication shortages, limited facilities, and poor interactions with some healthcare providers. These findings highlight the need for better communication and greater attention to mothers’ needs in resource-constrained settings (7).

Globally, KMC has been acknowledged for its numerous benefits, particularly in North America, have implemented a practice change and integration of kangaroo care into their neonatal care routines, to reflect WHO recommendations, leading to a clinical increase in kangaroo care session frequency and increase in duration post-implementation (8).

There is a solid understanding of KMC and the proper involvement of NICU nurses in tertiary hospitals in Saudi Arabia. However, KMC should be implemented more broadly, emphasizing the need for staff and parent education and tackling significant challenges, such as hesitancy among caregivers and insufficient privacy in NICU environments (9). Al-Matary et al. conducted a cross-sectional study to evaluate parental perceptions of KMC in Saudi Arabia. The results indicated a generally positive perception of KMC among parents. However, the study also highlighted a significant difference in perceptions between parents of male and female infants, with parents of female infants reporting more favorable views. While parents in Saudi Arabia exhibit positive attitudes toward KMC, there is a need for enhanced awareness and support to facilitate its practice, especially among fathers and in addressing cultural considerations (10). These findings are consistent with the mixed-method systematic review that synthesized global evidence on how parents and healthcare providers perceive, experience, and understand KMC. It revealed that while health care providers generally appreciate and support KMC, a portion lack sufficient training to implement it consistently; simultaneously, parental awareness, especially among fathers, remains limited. Although overall attitudes toward KMC are positive, the review found scarce data on parental knowledge, underscoring the need for improved prenatal education, formal protocols, and staff training to institutionalize KMC effectively in NICU (11). A qualitative study from the Eastern Province of Saudi Arabia examined mothers’ experiences of preterm birth and neonatal care. Mothers described significant emotional strain, particularly feelings of fear and uncertainty during their infants’ NICU stay. Supportive communication with healthcare providers and involvement in their infants’ care were identified as key factors that helped mothers cope. However, cultural competency training should be a core requirement for nurses to improve their understanding of the diverse needs of families during this sensitive period (12).

Despite cross-sectional studies showing positive attitudes toward KMC (10, 13), the practice of KMC remains low in hospitals across Saudi Arabia (13). Contributing factors include limited awareness and familial reluctance to initiate KMC (10). These challenges highlight the need for qualitative research to explore parental knowledge and experiences, and to understand parental barriers and facilitators of KMC in NICUs. Therefore, this qualitative study aimed to assess parental knowledge and experiences regarding kangaroo care in Saudi Arabia and explore the factors influencing their decisions to practice it.

## 2 Materials and methods

### 2.1 Study design and setting

This study employed a descriptive qualitative design to explore the knowledge and experiences of parents practicing kangaroo care (KC) with their preterm infants. The study was conducted in the Neonatal Intensive Care Unit (NICU) of a tertiary hospital in Riyadh, Saudi Arabia. The NICU provides care specifically for preterm and high-risk neonates and acts as a main referral center for the region. The unit is supported by a team of neonatologists, nurses, and other health professionals. Kangaroo care and family-centered practices are regularly part of the care offered to infants and their families.

### 2.2 Participants and sampling

A purposive sampling strategy was used to recruit 18 participants. The sample size was determined by approaching the saturation point (14). In this approach, the sample size is determined by continuing data collection until thematic saturation is reached, that is, when successive interviews yield no new concepts or insights, suggesting that additional participants would not contribute further to the depth or breadth of the findings.

Eligible participants were biological mothers and fathers, aged 20–50 years, who had engaged in KC at least twice with their preterm infants. Eligible infants were moderate to late preterm (32–37 weeks’ gestational age), admitted for ≥2 days with stable vital signs.

Exclusion criteria included: (1) congenital malformations or genetic disorders that prevented safe KC, (2) secondary admission to the NICU, and (3) parental cognitive or communication barriers.

### 2.3 Data collection

Data was collected between March and April 2025 through semi-structured, individual interviews, conducted either in a private hospital room or by phone. A kangaroo care coordinator first approached the parents, and after they gave preliminary approval, the researchers explained the study and informed them of consent. Data collection began when the participants provided informed consent. Participants were given a choice between face-to-face or teleconference interviews. 16 interviews were conducted by teleconference, and two face-to-face interviews. Interviews were conducted in Arabic, and selected themes were translated into English using a forward–backward translation process (15). Any discrepancies were resolved collaboratively within the research team.

The interview process was conducted by two moderators, who had the experience and the necessary training to conduct qualitative studies. The authors facilitated each interview to ensure depth and comprehensiveness. The interview guide was developed from literature (Appendix 1) and progressed from general to specific prompts, exploring awareness of KC, personal experiences, and perceived support from healthcare professionals. Interviews lasted 30–60 min, were audio-recorded and transcribed verbatim, and continued until data saturation was reached. To ensure ethical standards, participants received a detailed information sheet, provided written informed consent, and were reminded of their right to withdraw at any time. Confidentiality and privacy were maintained throughout.

### 2.4 Data analysis and rigor

Data was analyzed using Braun and Clarke (16) six-phase reflexive thematic analysis. The data analysis process began with engagement with the raw data, listening to the interviews, and then reading each transcript twice. Next, the authors performed initial inductive coding. They used a table to organize the quotes and codes and write notes. After the initial coding, the authors met to reach a consensus on the coding. It was mostly semantic coding, with some latent coding. The authors then explored categories and themes across the codes. Next, they revised the suggested themes with the data extracts. Some of the data was re-coded until coherence was achieved. Once each author was satisfied with the analysis and the themes, they met again to discuss and reach a consensus on the final analysis.

Rigor was maintained through credibility, transferability, dependability, and confirmability. Strategies included: (1) credibility was strengthened through triangulation by using multiple data sources (2) comprehensive documentation of methods to support transferability, (3) audit trails of audio files and field notes to ensure dependability, (4) collaborative review of coding and themes to strengthen confirmability, and (5) regular team discussions to refine interpretations.

### 2.5 Ethical considerations

Ethical approval was obtained from the Institutional Review Board of Princess Nourah University (log# 25–0106) and from the participating hospital (log# 25-086E). A pilot interview was conducted to refine the interview guide. Participants’ identities were protected using pseudonyms (e.g., P1, P2). All demographic information, transcripts, and recordings were de-identified and securely stored. The study followed the Declaration of Helsinki (2013 revision) (17).

### 2.6 Researcher reflexivity

The primary analysis was conducted by a female researcher, and a mother of two children. Her personal experience of pregnancy complications and emergency delivery during the COVID-19 pandemic, although resulting in a full-term birth, has shaped her sensitivity toward the challenges mothers face during the perinatal and neonatal period. This background may have enhanced her empathy and attentiveness to mothers’ accounts of vulnerability, uncertainty, and resilience in the NICU context. Professionally, the researcher is a qualitative researcher with a longstanding interest in maternal and child health, and an active advocate for child rights. Her previous scholarly work on domestic violence and childhood trauma may have influenced her interpretive lens, particularly in identifying themes related to emotional distress, coping strategies, and the broader social determinants of maternal experiences. Additionally, her extensive international exposure and multicultural practice across different countries provided her with a broader perspective on cultural diversity in maternal care practices. This may have facilitated openness to varied narratives and reduced the risk of imposing culturally narrow interpretations on the data. At the same time, the researcher acknowledges that her dual identity as both a mother and a child health advocate could have introduced interpretive bias, particularly a tendency to focus on themes of maternal strength and advocacy. Throughout the analysis, she engaged in reflexive questioning and critical discussions with co-researchers to ensure that her positionality enriched rather than constrained the interpretation of mothers’ voices.

## 3 Results

**Figure 1:**
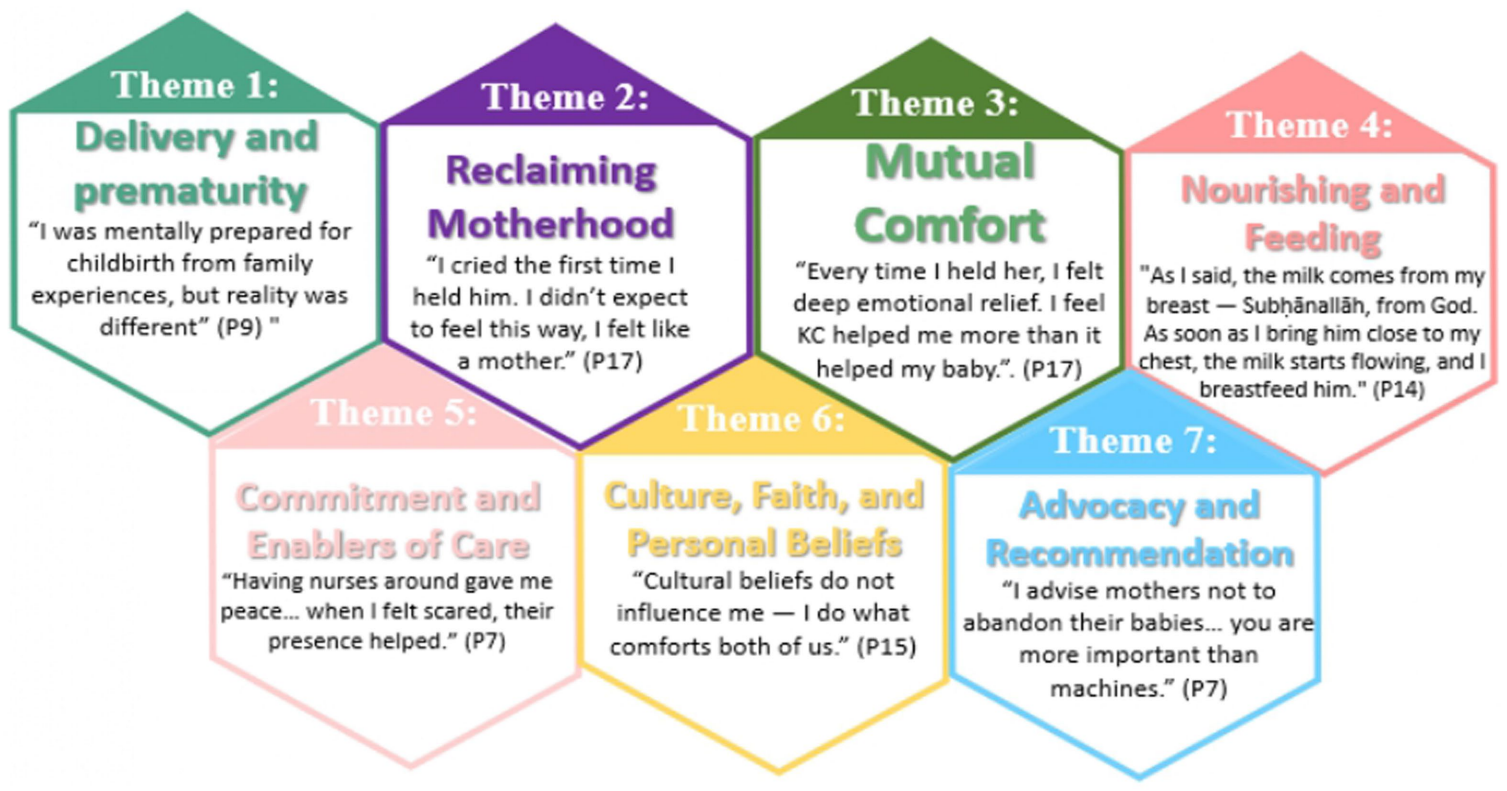
Themes of participants’ knowledge and experience of KC for pre-term infants in the NICU.

This study explored the perception and lived experience of 18 married mothers, aged 28–40 years, who had pre-term infants (Table 1). Their infants were admitted to the NICU for various reasons, and these mothers were encouraged to practice KC with their infants. The thematic analysis of the 18 interviews produced seven themes, and five sub-themes. In these themes, we capture the motherhood journey through KC and reflect on various aspects such as emotional healing and social support (Figure 1; Table 2).

**Table 1:** Participant characteristics among parents of preterm infants in NICU (*N* = 18).

Parents’ gender	
Female	18
Male	0
Nationality	
Saudi	18 (100%)
Non-Saudi	0
Age (years)	(28–40)
Marital status	
Married	18 (100%)

**Table 2:** A summary table that presents seven and five sub-themes.

Theme	Description	Subthemes
1. Delivery and prematurity	Mothers’ experiences surrounding unexpected preterm birth and early NICU admission	Not applicable
2. Reclaiming motherhood	Mothers’ journey of emotional healing and identity reconstruction through KC	Not applicable
3. Benefits of kangaroo care: mutual comfort	Perceived benefits of KC for mothers, infants, and bonding	For mothers: emotional relief, reduced guilt, increased confidence For infants: calmness, improved oxygenation, faster recovery For bonding: sensory connection, attachment, infant recognition
4. Nourishing and feeding	The role of KC in breastfeeding and maternal competence	Not applicable
5. Commitment and enablers of care	Factors influencing sustained engagement in KC	Internal motivation and commitment: responsibility, emotional drive, persistence External support system: – Medical staff (education, reassurance, logistics) – Husband (emotional/logistical support, variable participation) – Extended family (childcare, emotional and practical support)
6. Culture, faith, and personal beliefs	Cultural and spiritual contexts shaping KC experiences	Not applicable
7. Advocacy and recommendation	Mothers’ reflections and recommendations for future practice	Not applicable

### 3.1 Theme 1: delivery and prematurity

In this theme, the mothers narrated their stories from the beginning, before starting the KC practice. They described the painful experience of unexpected preterm delivery, the need for NICU admission, and the early forced separation from their babies. This theme captures a unique layer of emotional, psychological, and physical vulnerability that the mothers carry on their shoulders. Across the interviews, many mothers vividly described their shock due to the unexpected medical complications, high anxiety due to the early separation from their infants and NICU admission, and general emotional trauma. They described being traumatized and feeling helpless.

Some mothers dealt with the combined trauma, such as preterm delivery after infertility, and a complete lack of knowledge about prematurity. One participant said:

“My delivery was premature, and completely unexpected labor just started suddenly, and I gave birth. I had waited nearly eight years to get pregnant, and the birth was difficult. It was my first child, and I had no experience at all—not with childbirth and certainly not with preterm babies.” (P17)

Also, mothers had their internal struggles due to the mismatch between expectation and reality after preterm delivery; One participant said,

“I was mentally prepared for childbirth from family experiences, but reality was different.” (P9)

Another participant described her psychological status by saying that:

“It was obvious that I was scared right after giving birth… My emotional state was very unstable.” (P2)

Also, physically, mothers were weak and suffering. One mother described:

“Because I had a C-section, it was difficult for me to walk during the first few days.” (P16)

Some mothers were dealing with past traumatic experiences with preterm birth:

“My first experience was hard—I lost the first baby.” (P11)

Guilt was also a prominent emotion expressed by mothers following premature delivery, as many questioned whether they had done something wrong. One participant reflected deeply on this:

“I kept wondering, what mistake did I make? Could it really be because I did not change my lifestyle? I lived normally, whether pregnant or not; my days looked the same. So, I asked myself, “Should I have been more careful? Should I have gone out less, stayed home more?” But then I had a preterm birth, out of nowhere and without any clear reason. That’s when guilt started to consume me. I thought, “Maybe I pushed myself too hard… What did I do wrong?.” (P6)

These quotes highlighted the psychological burdens that weighed on the mothers’ hearts before KC even began; such a burden might have contributed to their internal motivation to practice KC, as they might have considered it an emotional refuge from all the early emotional shock.

This suggests that KC functioned not only as a caregiving practice but also as a reparative mechanism, enabling mothers to reinterpret traumatic birth as the starting point of a renewed maternal role. Within an attachment framework, the shift from helplessness to agency reflects how skin-to-skin contact accelerates the re-establishment of disrupted maternal identity.

### 3.2 Theme 2: reclaiming motherhood

In this theme, mothers described their journey in reclaiming motherhood and healing from the early trauma, mostly done through KC practice. However, this journey was neither easy nor straightforward. It took a long time for the mother to overcome the emotional toll. They moved through different stages and sometimes went back and forth between them. All mothers described that they were very anxious and fearful at first sight of their infants hooked on wires and sometimes ventilators, the atmosphere was full of medical uncertainty, and in some cases, they did not feel that these were their babies. Then they were the moment of the first touch and skin-to-skin contact, which came after a different time frame, ranging from a few days to months. After practicing KC with continuity, they reported an emotional shift from fear and discouragement to deep attachment and pure joy.

Almost all mothers reported that the first stage of this journey was fear and hesitation. These emotions were primarily triggered by the infant’s low birth weight and the medical wires connected to the infant’s body. When mothers were introduced to the KC by the medical team, it was not an easy task to do due to various reasons, for example, (P1) explained:

“They [the babies] were hooked to wires, and I was terrified… I did not want to touch them… afraid I might hurt them.” (P1)

In some cases, the fear was due to a lack of knowledge. For example:

“Honestly, I did not know about it. I was afraid I’d infect him.” (P3)

After this stage, the mothers clearly described the powerful emotions during the first session of the KC practice, which they marked as the first moment of motherhood. According to many mothers, the first KC session was a turning point where they felt a true sense of belonging and healing from early birth trauma.

This turning point can be understood as a reconfiguration of maternal identity: KC transforms mothers from peripheral observers of a highly medicalized NICU space into active participants. This aligns with theories of maternal role attainment, where embodied caregiving practices anchor women’s transition into motherhood, particularly under conditions of prematurity.

For example,:

“I did not feel like I had given birth until I held her… before that I only saw her through the glass.” (P4)

The first touch was always described as a decisive moment.

“I cried the first time I held him. I did not expect to feel this way; I felt like a mother.” (P17) “First time I held her was after a month—I cried from both joy and fear.” (P16) “The first time I could smell him and hold him—it was a wonderful feeling.” (P13)

Considering that this moment is sometimes delayed due to various medical reasons. The mothers referenced factors that helped decide the readiness for the KC practice, such as weight gain and removal of the ventilators. For example:

“I was afraid at first, especially in the first month… my daughter was very weak… weighed around 700 grams. The first time I held her, she weighed about 1.3 kg… it was about a month after birth.” (P4)

After this emotional roller coaster came the third stage, where mothers started to settle, have emotional stability, adjust to the environment, and feel used to the KC practice. At this stage, all mothers discussed how KC empowered them and shifted them from passive observation to active care. They now have a sense of usefulness and are sure they are helping their babies improve in a setting full of medical uncertainty. That does not deny the fact that they still feel some fear and anxiety, but they have learnt how to overcome it for their infants’ benefit. For example,

“Initially there was fear and anxiety, but it changed with time and practice.” (P2)

Also, another participant added:

“At first, I was scared of the oxygen equipment, but I got used to it.” (P12)

The progress in maternal confidence was shown with (P6) when she said:

“At first I was frozen from fear… later I learned how to move and handle him confidently.” (P6) Also, another mother described it as: “At first, I was afraid to touch him — I would recite the Quran from a distance. After some time, I started holding him.” (P12)

And at the end of the journey all mothers reported the dramatic change in their feelings toward the KC and how did they become very confident in doing it, furthermore they are very keen and eager about the sessions, one participant explained it while laughing:

“I kept asking for kangaroo care, even teasing them [the nurses] — I wanted to do it.” (P1) Another participant beautifully described it by saying: “I truly felt a different sense of motherhood… there was harmony.” (P2)

In general, many mothers did not perceive KC as a medical recommendation; instead, they marked it as a transformative experience that made them feel like true mothers of actual babies.

### 3.3 Theme 3: benefits of the KC practice—mutual comfort

In this theme, we reflect on the mother’s perceived benefits of the KC. Many benefits were perceived from the KC sessions, some for the mothers, some for the infants, and some were mutual for the bonding.

### 3.4 For the mothers

In this subtheme, we explored the benefits of the KC session to the mothers themselves. From the mothers’ perception, KC gave them a sense of power, purpose, and competence. One mother described the KC session as emotional release and total emotional immersion:

“It’s a break because there’s no effort or exhaustion. Just me and my baby… a moment to disconnect from the world.” (P3)

KC was described as psychological buffer tool for the guilt feeling, one mother said:

“the Kangaroo care distracted me from guilt.” (P6)

Also, the KC was considered as coping and soothing mechanism, for example one participant declared clearly:

“Every time I held her, I felt deep emotional relief. I feel KC helped me more than it helped my baby.” (P17)

These statements reveal KC as a coping framework: by reframing maternal suffering through physical closeness, mothers constructed meaning and emotional stability. This indicates that KC served not only as care for infants but as a resilience-building mechanism for mothers themselves.

Emotional uplift was also reported as a benefit of KC practice:

“I saw my baby improve and felt hopeful.” (P7)

Many mothers credited KC as a tool that boosted their confidence in caring for fragile and vulnerable infants. This confidence prepared them for home care after discharge. One participant said:

“It helped me get used to how to carry him in the future—when he comes home. It taught me how to hold him, how to care for him, and how to handle everything. It really helped me.” (P6)

### 3.5 For the infants

In this subtheme, the mothers described their perception of the benefits of KC on their infants’ well-being. To varying degrees, all of them believed that KC benefited their infants. They observed tangible, minor improvements that they attributed to KC, such as:

General improvement:

“After each KC session, I felt she was stronger… even the doctors said she improved.” (P6), and “I linked my daughter’s improvement to kangaroo care.” (P4)

Calmer behavior:

“She calmed and slept when I held her.” (P16), “When I saw her, I read Quran and breastfed her… this comforts the baby.” (P8)

Better oxygen saturation:

“At the moment I held her, the oxygen level went up—they saw it on the monitor; her oxygen saturation increased.” (P17)

Speedy discharge:

“She was discharged earlier than expected.” (P16)

Emotional security:

“The baby feels safe when we hold her on our chest.” (P18)

### 3.6 For the bonding

In this subtheme, the mothers described the mutual benefits for them and their infants during the KC practice sessions. Most mothers felt they had greater connections and bonds with their infants due to the skin-to-skin contact. One mother said with pride:

“KC strengthens the bond… he feels warmth, safety, and calms down.” (P3)

The deep attachment was always referenced as a result of the KC practice:

“I became very attached to her after doing KC.” (P18)

Also, from the mothers’ perspective, KC humanized their infants as they started interacting with them. Mother-infant sensory connection was always referenced. Mothers were confident that their infants would begin recognizing them due to the close physical contact. For example:

“She grabbed my hand and looked at me like she knew me.” (P17) Also, “She grabbed my chest with her hand… it felt so beautiful.” (P10)

“I felt she recognized me when she smelled and touched me… motherly intuition is different.” (P10) “He keeps looking at me… once he even puts his hand on my cheek.” (P2) Stronger feeling Bonding through senses was added: “Smelling him made me feel he was truly mine.” (P13)

From an attachment perspective, these sensory interactions highlight how KC accelerates the biological and emotional attunement between mother and infant, grounding early bonding in both physiological cues and maternal identity reconstruction.

### 3.7 Theme 4: nourishing and feeding

In this theme, the mothers described a recurrent perception that KC significantly helped with milk production. In most of the narratives, KC was deeply intertwined with feeding. Nearly all mothers reported that the practice of KC increased milk production or made breastfeeding easier. Furthermore, all of them marked this as a significant observation. For example,

“At first, I had no milk, but after kangaroo care it gradually came. I’d bring the pump with me.” (P1) “Milk production increased after holding and smelling him.” (P2) “I noticed more milk after starting KC.” (P17) “After starting kangaroo care, I noticed an increase in milk production.” (P11)

Some were surprised by the immediate milk letdown upon holding the baby skin-to-skin. For example,

“As I said, the milk comes from my breast—Subḥānallāh, from God. As soon as I bring him close to my chest, the milk starts flowing, and I breastfeed him.” (P14)

And the first direct breastfeeding experience was marked as a joyful moment:

“They told me to nurse her in the room… first time she latched; I was overjoyed.” (P10)

Also, most of the mothers were very grateful that the hospital had a rigorous policy regarding natural feeding, one participant said:

“They care deeply about breastfeeding.” (P12)

They also encouraged them to pump milk at home and bring it with them during the KC session, so even for the infants that were too weak to be breastfed, they were fed their mother’s milk. For example:

“The lactation specialist there spoke to me and said it’s okay even if nothing comes out—just try every two hours. So, I kept trying until eventually the colostrum came out, and I gave it to them right away.”(P12)

Furthermore, frequently, the mothers reported receiving calls asking for an extra milk supply. And these requests were always received with love and enthusiasm. For example:

“I used to pump milk at home and deliver it to them daily. One day, about six hours after I returned from a visit, they called and told me that they had run out of milk, so I had to go back and bring more.” (P16)

In general, it is clear from the mothers’ experiences that breastfeeding was essential in shaping their relationship with their infants during critical care. It also helped them strengthen their sense of motherhood during this challenging period. The mothers unanimously agreed that KC involving skin-to-skin contact was instrumental in increasing milk production. Similarly, for mothers who were unable to breastfeed and resorted to pumping milk to feed their premature babies, this was an ideal option, helping them feel connected and included at a time when medical conditions prevented breastfeeding. This underscores the dual role of KC in sustaining maternal competence: it meets the infant’s nutritional needs while simultaneously reinforcing mothers’ embodied caregiving identity. Through a self-efficacy lens, breastfeeding linked to KC can be seen as a concrete achievement that validates mothers’ agency in an otherwise medically dominated environment.

### 3.8 Theme 5: commitment and enablers of care

In this theme, the mothers spoke truly about the factors influencing their decisions to practice KC. And their feelings toward the leading enablers that allowed them to practice KC consistently.

This theme is divided into two subthemes: internal and external enablers.

### 3.9 Internal motivation and commitment

In this subtheme, mothers explained that their primary motive to practice the KC came from inside; they felt responsible and willing to do anything to speed up the recovery. One participant explained:

“I was truly scared for her…. She was tiny and thin. I just wanted her to grow well in any possible way, so I followed everything they told me to do.” (P18)

Mothers emphasized that it all comes from internal motivation, and no external pressure was there. Mother said:

“There wasn’t pressure… I was excited… I did not feel stressed.” (P5)

Some mothers felt that the KC practice compensated them for the initial separation due to preterm delivery. And that showed that sometimes attachment might be formed through separation. The mother said,

“Maybe because they took her from me at birth, I became very attached, never missed a day.” (P10)

Also, KC practice helps mothers show sympathy to their infants. One mother said:

“I used to feel that she was helpless and all alone… I just wanted to let her feel that I was there for her, that I had not left her.” (P16)

In general, all mothers showed a very high level of engagement, for example,

“I visited twice daily—morning and evening.” (P14)

This engagement has positive reinforced feedback for further engagement, explained:

“Every time I saw her improve, I got more motivated to visit.” (P18)

Mother showed persistent commitment despite challenges and maternal burden, such as:

“I worked remotely and kept my laptop next to me… I’d pause work, then continue. I worked 12 h instead of 8—what mattered most was seeing my baby.” (P1)

The same commitment was shown with another mother,

“I was under stress from other kids and moving houses. Despite that, I never skipped a visit.” (P18)

Also, another participant indicated that despite her having another household responsibilities and burdens, she always felt that her infant comes first, and she is the priority, and hence this mentality helped her in overcoming all the burdens and challenges and made her committed to the daily visit and KC practice, explained:

“I visited her daily despite distance and home responsibilities.” (P18)

Another mother also mentioned that she resigned from her job to fully dedicate herself to caring for her pre-term infant. She said:

“I used to work, but when I started feeling overwhelmed, I stopped working until things settled down, until I felt better and ready to return to work.” (P2)

Another mother whose baby was admitted said:

“I went daily, sometimes twice a day, and did kangaroo care for hours.” (P6)

Even distance did not limit KC’s commitment,

“I lived an hour from Riyadh, but still went daily.” (P7)

These testimonies reveal that mothers during this period cared for nothing but their premature babies. Despite the challenges and obstacles they faced and the responsibilities they shouldered, they left everything behind and focused solely on their children’s needs. This stance stems from a deep sense of their children’s need for them. As one mother explained:

“I did not care about the house… she really needed me.” (P8)

This persistence illustrates how internal motivation interacts with maternal role theory: caregiving became central to mothers’ self-definition, often overriding competing responsibilities such as work or childcare.

### 3.10 External support system

In this subtheme, the mother emphasizes that although the intrinsic desire motivates them to practice KC, the external enablers make the practice sustainable. The key external enablers, who attributed the success of practicing KC, were similar across all mothers’ experiences: the medical staff, the husband, and the extended family.

#### 3.10.1 The medical staff

The medical staff were always referred to as the first source of encouragement, initiation, hands-on support, and trustworthy information about KC. For example,

“The nurse encouraged me and said, just try it—you’ll see the difference.” (P4), and another mother explained: “They encouraged skin-to-skin and explained its benefits.” (P13)

From the mothers’ experiences, they expressed that they felt empowered by the professional support they had received. As explained:

“The specialist inspired me… she said: no one can deny you your baby, you are his medicine.” (P7)

Also, mothers explained that what encouraged them to overcome the initial fear was knowing the medical benefits of the KC from the nurses, she said:

“They [the nurses] told me the mother needs to hold the baby—it helps the baby feel you, and it stimulates milk flow.” (P1)

Psychological support was marked as one of the main actions taken by the medical staff. And that showed the fragility of the mothers during the postpartum period and in the medical setting. Emotional reassurance from staff was frequently mentioned. For example,

“Having nurses around gave me peace… when I felt scared, their presence helped.” (P7) And “The nurses constantly reassured me…. their emotional support was powerful” (P2)

Also, the participant explained that the general climate at the medical facility was very supportive of the KC practice. One participant said:

“Doctors, nurses, even med students, all helped… They welcomed me and asked about me.” (P6)

Logistical and facility support in creating a comfortable KC setup was mentioned as an enabler also, for example,

“The hospital gave me privacy with a curtain and pillow support.” (P12)

Even small gestures of logistical and physical support were highly appreciated. One mother recalls saying,

“The staff would bring me a chair… help with a cover… sometimes offered water or juice.” (P5)

One mother said,

“They were absolutely wonderful—whatever the mother needed, they responded to it immediately.” (P1)

And in all aspects, mothers speak about the medical staff with deep gratitude and appreciation.

This highlights how relational trust with medical staff operated as an external scaffolding for maternal confidence, suggesting that institutional support is not merely facilitative but constitutive of mothers’ ability to sustain KC.

#### 3.10.2 The husband

Paternal involvement in KC, whether active or limited, was consistently perceived positively by mothers. While the degree of participation varied, mothers expressed appreciation for their husbands’ presence and encouragement, highlighting their significance in reducing stress and reinforcing their confidence in caring for a pre-term infant.

Many fathers actively practiced KC; they visited the NICU frequently and held their infants in skin-to-skin contact. And from the mothers’ perspective, they believed that fathers who practiced KC were emotionally connected and bonded to their infants. For example,

“My husband enjoyed doing it KC— he said he felt like a real father… like there was a bond between him and the baby.” (P1) “My husband accompanied me to every NICU visit… never let me go alone. He held her on the third or fourth day — he was nervous but did it.” (P10) “Her father would place her against his chest, and I truly felt that she was improving day by day” (P18)

Another participant said she encouraged her husband to practice the KC:

“My husband was hesitant—he was afraid—but I was the one who supported him. I used to encourage him, telling him, “Hold him, hold him, you’ll feel it,” and he would say, “I’m scared.” I would reassure him, saying everything was fine. (P2)

Some fathers were considered supportive in logistical support, but they did not practice KC. They provided the needed emotional support and transportation, for example:

“My husband came and entered with me but did not hold her.” (P16)

The lack of active participation of fathers in the KC session was attributed to several reasons. One mother highlighted the barriers to father participation in the KC sessions, she said:

“My husband did KC twice, but was discouraged due to smoking… he even tried to stop smoking” (P4)

However, some mothers reported father disengagement due to gendered perception, for example:

“My husband refused to do kangaroo care… he said I should because the baby was in my womb.” (P6)

Some mothers attribute the fathers’ lack of involvement in care to the lack of motivation from the medical staff.

“He did not practice it himself because the staff did not ask him to do it. They prioritize the mother more than the father.” (P17)

Another reason cited by the mother was extreme fear of harming the baby. One mother explained:

“When his father saw me holding the baby, he said, ‘Do not hold him, just leave him.” I told him, “It’s okay—I’m his mother. I do not think I’m going to harm him.” But he kept saying, “Maybe you are carrying something.” He was afraid and said, “I do not want to hold him because I’m scared, I might pass on germs. It’s up to you, but I do not want to risk doing anything to him.” (P11)

Regardless of the shape of the support provided by the husband, all mothers agreed that it is a crucial element for the sustainability of the KC practice by the mother. One mother explained it clearly:

“The mothers had absolutely no issue—they would follow instructions without hesitation. If you told a mother, It’s medically necessary to walk through fire, she would do it without question or argument…But if the father is not supportive, she cannot sustain it.” (P6)

#### 3.10.3 The extended family

The role of the extended family was marked as a key element in supporting mothers during their NICU and KC experiences. The family’s involvement took various forms.

Caring for older children was one of the main supports provided, as mothers felt comfortable and assured that their older children were being cared for and in safe hands. For example:

“I used to leave my older child with my mother so she could take care of him, as he was still around 11 months old.” (P11)

One participant described her family learning about KC and involvement and trying their best to provide educational materials; She said,

“My family sent me videos — showing how to hold the baby in the chest.” (P4)

Other forms of support that were reported are being enthusiastic about the KC and providing continued encouragement for practicing, for example:

“Honestly, they encouraged me. They would ask, ‘Did you go to the hospital today? Why not?’ and inquire about my child’s health.” (P12)

Physical and logistical support from family was also consistently reported, such as:

“I had a C-section, and it was hard to move… my mom and sister accompanied me.” (P7)

Another participant said:

“My family woke me up to attend doctor rounds” (P6)

Also, the family was counted as an essential source for providing emotional backing. For example,

“I was under emotional pressure, but family support helped me through.” (P11)

And another participant said:

“I often cried — my family just reassured me she’d be okay.” (P16)

A few mothers also reported that the family was KC’s first exposure source. For example, one participant said that her sister had previously had a preterm infant and that she practices the KC:

“My sister, who also delivered early, I saw the difference and got excited to try it myself” (P2)

Furthermore, many mothers reported that their extended family holds cultural beliefs supporting KC, for example:

“My mom says to keep the baby close to his mother… it’s better mentally and physically.” (P2)

More importantly, after their experience, some mothers discovered that the practice of skin-to- skin contact with newborns was a common practice in their family for a long time but it was an unlabeled traditional ritual. For example, one participant explained:

“We practiced it but did not know the term… the word is hard to say, but we know what it is.” (P2)

### 3.11 Theme 6: culture, faith, and personal beliefs

In this theme, mothers navigated their journey with KC through social beliefs and personal values. A few mothers encountered negative social stigma and misconceptions regarding pre-term infants and KC, for example:

“There are false beliefs that preemies are disabled, which is completely wrong.” (P4).

Another negative view included discouraging bonding with fragile infants who might pass away. One participant explained very well,

“There’s a cultural belief in the community around me that says, ‘Do not get attached—you never know.” They treat it like you are playing roulette: 50% chance of loss, 50% chance of winning. So why do you take the risk? They say, “If the baby survives, fine—go see them. Otherwise, just leave them at the hospital. If they pass away, you will not have gotten emotionally attached as a mother.” (P6)

Most mothers generally did not face social resistance to the KC practice. Those who faced some issues reported that they had rejected these views entirely and overcame them through KC. One mother firmly shared her experience:

“I would not allow anyone to continue talking like that around me. I completely rejected it. I’d say, “Do not say that to me. Medicine has advanced in every way—why should it stop progressing when it comes to a baby born early?” (P6) Another mother took a positive position by saying: “They told me not to get attached, but I could not help it… I felt I had to hold him.” (P16)

However, mothers reported that from their observation in the NICU, they believed that these negative views about the pre-term babies affected some parents and led to neglect, as they have noticed that some infants do not have visitors. For example:

“Some parents did not visit their babies… they said if he survives, we’ll come get him.” (P6)

Also, knowledge was a weapon mothers used to overcome hesitations due to the false social views.

“I was afraid to get attached because he was preterm… but when I understood the importance of KC, I visited daily.” (P7) Another participant added to that: “I was scared of people’s opinions, but I believed in the benefits of touch.” (P12)

Also, mothers emphasized autonomy in caregiving practice by firmly stating that:

“Cultural beliefs do not influence me — I do what comforts both of us.” (P15)

Here, KC becomes more than clinical practice; it emerges as a counter-narrative against cultural fatalism. By reclaiming emotional attachment as a moral and spiritual responsibility, mothers integrated caregiving with resilience rooted in faith. This positions KC as both a biomedical and culturally embedded practice.

### 3.12 Theme 7: advocacy and recommendation

In this theme, we summarized the mothers’ home messages from their experience. All mothers were generally very grateful that they were introduced to KC. However, they raised two crucial points: first, that teaching about KC should have started before birth for both parents, not after, and secondly, the mothers suggested that more advocates and educational efforts should be dedicated to KC.

At the first point, mothers explained that the postpartum period is very overwhelming, and prior preparation for the KC sessions would have been invaluable. They recommended that KC be included in antenatal care sessions. One participant said,

“I wish we were told about kangaroo care before delivery, not after.” (P17)

The same recommendation was repeated many times:

“I wish pregnant women were educated about KC beforehand.” (P16)

And peer exposure before delivery was suggested as emotional prep, for example:

“I’m saying this from experience. It was my first baby, and they told me at 34 weeks that I’d be having a C-section. So, I was admitted. And yes, it might be a little challenging to implement, but if they could arrange a meeting between a mother of a pre-term baby and someone who has just been told they’ll deliver early, it would really help. Seeing a mother who’s lived that experience gives you hope.” (P7)

Secondly, the mothers suggested more advocates and educational efforts should be dedicated to KC, as many in their community and social circle had never heard about it before; they said:

“Some mothers lack awareness… they need someone to educate them.” (P3) And “I suggest educational videos about KC.” (P4)

Those participants sent strong encouragement statements for the future mothers; they all emphasized the consistent presence of parents. For example,

“I hope every mother stays close to her baby… that’s the bond of love.” (P8) And “I advise mothers not to abandon their babies… you are more important than machines.” (P7) Another piece of advice, “Be as close to them as possible… even for a short time, they feel your love.” (P5)

“I advise any mother to hold her baby and recite the Quran.” (P12)

“I shared my experience with everyone and told them to do the same.” (P18)

Lastly, calls for stronger father education and involvement were made by the majority of the participants, for example:

“I advise mothers and fathers… It makes a difference in parenting. Any mother who wants to be close to her baby should not hesitate… and the father should be encouraged.” (P2) And “I wish all fathers were educated about kangaroo care and how to handle their baby.” (P10)

The strength of the recommendations resulting from the mothers’ experiences clearly shows that the benefits they gained from KC left them highly satisfied with the experience. All narratives strongly advocate for KC integration in antenatal education and neonatal care, and better education for both parents equally.

## 4 Discussion

This qualitative study aimed to assess parental knowledge and experiences regarding kangaroo care in Saudi Arabia and explore the factors influencing their decisions to practice it. The study identified seven themes and five subthemes that deepen understanding of the experiences of mothers practicing KC in the NICU, illustrating its role as a transformative intervention that extends far beyond a clinical technique.

The study’s findings highlight that mothers endured painful and unexpected preterm deliveries, leading to heightened anxiety and overwhelming feelings of guilt. However, in line with prior research, introduction to KC demonstrated remarkable benefits: it alleviates maternal distress following preterm birth, fosters stronger emotional bonding with infants, supports breastfeeding, and reinforces maternal identity and confidence (18–20). Notably, Ionio et al. and Kurt et al. highlight KC’s ability to restore emotional balance through oxytocin release and bonding (18, 21). This suggests that KC functioned not only as a caregiving practice but also as a reparative mechanism, enabling mothers to reinterpret traumatic birth as the starting point of a renewed maternal role. Within an attachment framework, the shift from helplessness to agency reflects how skin-to-skin contact accelerates the re-establishment of disrupted maternal identity (22).

Equally important, KC was experienced as a process of empowerment. As in Mu et al., Vogl et al., and Sjömar et al. (19, 20, 23), participants in this study described initial fear and hesitancy, followed by growing confidence and competence as they engaged in KC. Additionally, a study investigating the experiences of parents engaged in KC revealed that both mothers and fathers experienced increased confidence in their parenting abilities over 7 days (24). Similarly, other research has demonstrated that mothers who practiced KC exhibited reduced anxiety levels and enhanced confidence in their ability to care for their infants following discharge (25). This progression highlights the intervention’s capacity to transform mothers from passive observers into active caregivers, affirming their identity and reinforcing family-centered models of neonatal care.

The study also reinforces the dual benefits of KC for both maternal and infant well-being. Maternal accounts of emotional comfort, reduced guilt, decreased maternal stress, and improved confidence echo the psychosocial healing documented in previous research (19, 20, 26). At the same time, mothers observed visible improvements in infant health, such as calmer behavior, better oxygen saturation, and weight gain—consistent with (19, 20, 26–28). Together, these findings emphasize KC as a mutually beneficial practice that simultaneously nurtures the physical stability of infants and the emotional resilience of mothers.

The role of KC in breastfeeding further underscores its integrative impact. In line with previous research (26), mothers reported that KC enhanced milk production, facilitated lactation, and made feeding more manageable. Beyond physiology, these moments were imbued with symbolic meaning: providing milk was seen as a vital contribution that reaffirmed maternal agency in the NICU. This underscores the dual role of KC in sustaining maternal competence: it meets the infant’s nutritional needs while simultaneously reinforcing mothers’ embodied caregiving identity. Through a self-efficacy lens, breastfeeding linked to KC can be seen as a concrete achievement that validates mothers’ agency in an otherwise medically dominated environment. The encouragement provided by hospital staff and the structure of breastfeeding guidelines amplified these positive effects, highlighting the necessity of institutional support (29).

The findings under the “Commitment and Enablers of Care” theme focused on maternal commitment and internal/external enablers of KC that are consistent with regional and international literature. Mothers described intrinsic motivation rooted in emotional bond, a strong sense of responsibility, and the desire to aid their infant’s recovery. These findings align with Kurt et al., who reported that KC significantly enhanced maternal attachment, especially when implemented early and consistently (21). Enablers such as supportive healthcare staff, engaged fathers, and extended family played a vital role in sustaining KC. Consistent with regional evidence, Al-Shehri and Binmanee found that NICU nurses in Riyadh actively promoted KC and recognized its benefits for bonding and breastfeeding. Yet, barriers such as lack of privacy, staff workload, and paternal hesitancy mirrored the variations in father participation observed in our study (9).

Multiple mothers attributed their husbands’ limited participation in KC to several interrelated factors. Some fathers were hesitant due to fear of harming their premature infants, as they appeared small and delicate, which was a barrier well-documented in NICU settings (30). Other research cited cultural expectations and traditional gender roles, with caregiving often perceived as primarily a mother’s domain that aligned with broader findings on how rooted norms hinder paternal engagement (30). Additionally, a 2024 mixed-methods systematic review revealed that although healthcare providers often know KC, parents, particularly fathers, frequently lack awareness or structured invitations to participate, highlighting the need for more proactive provider support to ensure consistent involvement (11). Some mothers attributed fathers’ limited involvement in their infant’s care to a lack of encouragement or motivation from the medical staff. This points to the influence of institutional and staff-related factors on paternal participation. Hospital policies: such as, physical layout of the NICU, and staff practices, may also contribute to barriers that exclude fathers. Addressing these issues through policy changes and improved staff engagement could help create a more inclusive environment that supports greater parental involvement. These factors also help explain the absence of feasibility to obtain father-specific data in this study.

Some mothers described hesitancy toward early bonding with their preterm infants, often linked to concerns about uncertain survival. While this perception represents a barrier, it also highlights opportunities for supportive intervention. Healthcare providers may address these concerns through culturally appropriate counseling that acknowledges maternal fears, alongside peer support from mothers who have had positive experiences with KC. In addition, clear and targeted educational materials that address common misconceptions may help build maternal confidence and encourage early engagement.

Perhaps the most distinctive contribution of this study lies in its illumination of cultural and spiritual dimensions. While earlier work has acknowledged logistical or social barriers to KC (9, 10), the present findings reveal how faith, resilience, and resistance to societal stigma became integral to mothers’ persistence. KC was experienced not only as medical care but as an act of faith and cultural affirmation (31), where mothers actively rejected negative societal perceptions of fragile infants. This demonstrates that KC is embedded in broader cultural negotiations of motherhood, extending the existing literature by emphasizing how personal beliefs and spiritual practices shape caregiving experiences. Finally, mothers’ advocacy for wider KC implementation reflects the depth of its impact. Their assertion that maternal presence is “more important than machines” captures the essence of KC as irreplaceable human care. Calls for antenatal education and peer-led community support reinforce the need for earlier and more inclusive strategies that prepare parents, especially fathers, for kangaroo care. Integrating KC education into the existing Saudi maternal health system could include adding KC modules to routine antenatal classes, utilizing community health workers, and developing culturally tailored educational materials to be distributed during prenatal visits. These perspectives point to KC as more than a practice within hospital walls; it is a philosophy of care that requires structural, community, and cultural integration. By fostering an environment that supports early, culturally tailored education and family involvement, healthcare systems can improve parental empowerment and neonatal outcomes, aligning with the Sustainable Development Goals of ensuring healthy lives and promoting well-being for all ages (SDG 3) and reducing inequalities (SDG 10).

### 4.1 Limitations and future prospects

One of the main limitations encountered in this study was the need to conduct a total of 16 interviews via phone due to logistical constraints, which reduced the ability to capture non-verbal cues such as facial expressions and body language. However, all interviews lasted between 30 and 60 min, with no noticeable differences in length or emotional expression between teleconference and face-to-face interviews. To help compensate, careful attention was paid to changes in vocal tone and pitch to capture emotional nuances despite the lack of visual cues. Additionally, data collection from fathers was not obtained due to their restricted presence in NICUs, often due to work commitments and related scheduling constraints. While this difficulty mirrors practical constraints in involving employed caregivers, it nevertheless restricts the diversity of viewpoints included in the study and may affect the comprehensiveness of the finding. Traditional gender roles may have acted as a limiting factor by reducing fathers’ involvement, which in turn may have narrowed the range of perspectives reflected in the findings. The non-representation of fathers in the interview sample constraints the extent to which the study’s findings can be generalized Furthermore, the study faced a cultural limitation, as some mothers were hesitant to openly share personal emotions or detailed experiences related to KC, especially in a conservative societal context. This may have impacted the richness of certain narratives. However, the researchers made conscious efforts to create a safe and trusting environment during interviews, encouraging participants to express themselves freely. This was achieved using open-ended questions, empathetic listening, and reassurance that their stories were valuable and respected. In some cases, extra time was spent explaining the concept of KC to ensure participants had a clear understanding, which helped support more meaningful and informed discussions.

Integrating KC into clinical practice necessitates a multifaceted approach. Include KC education in antenatal care sessions. Pregnant women and fathers should receive structured information about KC during routine antenatal visits. This can help prepare them psychologically and practically for its application post-delivery, especially in cases of preterm birth. Offer personalized, one-on-one guidance by trained healthcare providers. Parents, especially those with premature infants, should be offered individualized education and hands-on training to ensure they understand how to perform KC correctly. Implement ongoing training and competency assessment for healthcare staff to promote and support father involvement in KC. Modify the physical setup of NICUs to allow for privacy, comfort, and accessibility, enabling prolonged skin-to-skin contact.

Future studies should be expanded across various hospitals and regions to gather broader, more representative data on KC experiences and barriers, particularly in diverse cultural settings. Additional qualitative research is needed to explore fathers’ involvement and experiences in KC, providing a more comprehensive view of family-centered care and identifying ways to better engage fathers in neonatal care. Longitudinal research should be conducted to evaluate the long-term health and psychological effects of KC on both infants and parents, offering deeper insights into its sustained benefits.

## 5 Conclusion

In conclusion, our study underscores that KC’s power lies not only in its clinical outcomes but in its ability to reaffirm the centrality of maternal presence in the survival and flourishing of preterm infants. A significant and unique finding of this study was the central role of cultural and religious values. Many mothers reported drawing strength from prayer and relying on their faith as a source of comfort throughout their KC journey, which is an aspect that has been largely underexplored in previous literature. However, participants expressed dissatisfaction with the timing of KC education, exclusion of fathers in KC participation, noting that it was often introduced only after delivery, a time when they felt emotionally overwhelmed and unprepared. They strongly advocated for the inclusion of KC education for parents, particularly fathers, during the antenatal period to enhance awareness and readiness. This approach not only benefits individual families but also contributes to the advancement of public health and pediatric care locally and globally.

## Data Availability

The raw data supporting the conclusions of this article will be made available by the authors, without undue reservation.

## Appendix 1

### Interview guide

1. What factors affected your decision to practice kangaroo care?

2. What support or assistance have you received during this process?

3. How do you think Kangaroo Care has influenced your bond with your baby? Prob question. How did you feel about it?

4. How has your family responded to your participation in Kangaroo Care? Prob question. What support or assistance did you receive from your family?

5. What challenges do you face in balancing Kangaroo Care with other responsibilities (e.g., work, family)? Prob question. What do you feel about it?

6. What are your cultural or personal beliefs that influence your participation in Kangaroo Care?

7. How do you manage feelings of stress or anxiety while practicing Kangaroo Care?

8. Would you recommend Kangaroo Care to other parents of pre-term infants? Why or why not?

9. What questions do you have about Kangaroo Care for pre-term infants? Do you have any other related needs?

10. What are your suggestions to help parents better participate in KC for preterm infants?

## References

[1] World Health Organization. Preterm birth. (2023). Available online at: https://www.who.int/news-room/fact-sheets/detail/preterm-birth (Accessed March 05, 2025).

[2] Ohuma E Moller AB Bradley E Chakwera S Hussain-Alkhateeb L Lewin A . National, regional, and global estimates of preterm birth in 2020, with trends from 2010: a systematic analysis. Lancet. (2023) 402:1261–71. doi: 10.1016/S0140-6736(23)00878-4, 37805217

[3] Als H McAnulty GB. The newborn individualized developmental care and assessment program (NIDCAP) with kangaroo mother care (KMC): Comprehensive Care for Preterm Infants. Curr Womens Health Rev. (2011) 7:288–301. doi: 10.2174/157340411796355216, 25473384 PMC4248304

[4] Sahni R Polin RA. Physiologic underpinnings for clinical problems in moderately preterm and late preterm infants. Clin Perinatol. (2013) 40:645–63. doi: 10.1016/j.clp.2013.07.012, 24182953

[5] Ramaiah R Jothishanmugam A Alshahrani SH Innocent Rani V Alshahrani BY Rajagopal Sambasivan L . Kangaroo mother care in-duced serum oxytocin facilitates prolactin and IL-10 among emergency Cesarean mothers. J Multidiscip Healthc. (2024) 17:2689–99. Published 2024 Jun 1. doi: 10.2147/JMDH.S444172, 38840703 PMC11152167

[6] Chen WY Wu YY Xu MY Tung TH. Effect of kangaroo mother care on the psychological stress response and sleep quality of mothers with premature infants in the neonatal intensive care unit. Front Pediatr. (2022) 10:879956. doi: 10.3389/fped.2022.879956, 35935377 PMC9354657

[7] Mimani Minuta W Lera T Haile D Badacho AS Bekele B Gezume Ganta A . Lived experience of mothers having preterm newborns in a neonatal intensive care unit at Wolaita Sodo University comprehensive specialized hospital southern Ethiopia: a phenomenological study. Res Rep Neonatol. (2023) 13:1–14. doi: 10.2147/rrn.s417173

[8] Beaumont L Mullaney D Eklund W DeGrazia M. Kangaroo Care in the Neonatal Intensive Care Unit-a Practice Change Initiative. Adv Neonatal Care. (2025) 25:129–37. doi: 10.1097/ANC.0000000000001252, 40085956

[9] Al-Shehri H Binmanee A. Kangaroo mother care practice, knowledge, and perception among NICU nurses in Riyadh, Saudi Arabia. Int J Pediatr Adolesc Med. (2021) 8:29–34. doi: 10.1016/j.ijpam.2019.11.003, 33718574 PMC7922834

[10] Al-Matary A Al-Matary M DelaCena S AlJohani E. Perception of parents in experiencing kangaroo care in Saudi Arabia. J Neonatal Nurs. (2023) 29:652–6. doi: 10.1016/j.jnn.2022.11.019

[11] Almutairi A Gavine A McFadden A. Parents' and healthcare providers' perceptions, experiences, knowledge of, and attitudes toward kangaroo care of preterm babies in hospital settings: mixed-methods systematic review. Birth. (2024) 51:690–707. doi: 10.1111/birt.12859, 39140585

[12] Alghamdi AA Althekrallah AY Sulayyil FAM Al Shawan DS. Mother’s lived experiences of preterm birth and neonatal care in the Eastern Province of Saudi Arabia: a qualitative study. Int J Africa Nurs Sci. (2025) 22:100843. doi: 10.1016/j.ijans.2025.100843

[13] Abdulghani N Cooklin A Edvardsson K Amir LH. Mothers' perceptions and experiences of skin-to-skin contact after vaginal birth in Saudi Arabia: a cross-sectional study. Women Birth. (2022) 35:e60–7. doi: 10.1016/j.wombi.2021.02.001, 33608236

[14] Guest G Bunce A Johnson L. How many interviews are enough? An ex-periment with data saturation and variability. Field Methods. (2006) 18:59–82. doi: 10.1177/1525822X05279903

[15] Chen H-Y Boore JR. Translation and back-translation in qualitative nursing research: methodological review. J Clin Nurs. (2010) 19:234–9. doi: 10.1111/j.1365-2702.2009.02896.x, 19886874

[16] Braun V Clarke V. Using thematic analysis in psychology. Qual Res Psychol. (2006) 3:77–101. doi: 10.1191/1478088706qp063oa

[17] World Medical Association. World medical association declaration of Helsinki: ethical principles for medical research involving human subjects. JAMA. (2013) 310:2191–4. doi: 10.1001/jama.2013.281053 24141714

[18] Ionio C Ciuffo G Landoni M. Parent-infant skin-to-skin contact and stress regulation: a systematic review of the literature. Int J Environ Res Public Health. (2021) 18:4695. Published 2021 Apr 28. doi: 10.3390/ijerph18094695, 33924970 PMC8124223

[19] Mu PF Lee MY Chen YC Yang HC Yang SH. Experiences of parents providing kangaroo care to a premature infant: a qualitative systematic review. Nurs Health Sci. (2020) 22:149–61. doi: 10.1111/nhs.12631, 31430017

[20] Vogl JL Dunne EC Liu C Bradley A Rwei A Lonergan EK . Kangaroo father care: a pilot feasibility study of physiologic, biologic, and psychosocial measures to capture the effects of father-infant and mother-infant skin-to-skin contact in the neonatal intensive care unit. Dev Psychobiol. (2021) 63:1521–33. doi: 10.1002/dev.22100, 33521969

[21] Yilmaz Kurt F Küçükoğlu S Aytekin Özdemir A Oğul T Türkön H Atay S . The effect of kangaroo care on cortisol levels and immune factors in breast milk. Dev Psychobiol. (2023) 65:e22402. doi: 10.1002/dev.22402, 37338250

[22] Mehrpisheh S Doorandish Z Farhadi R Ahmadi M Moafi M Elyasi F. The effective-ness of kangaroo mother care (KMC) on attachment of mothers with premature in-fants. Eur J Obst Gynecol Reprod Biol. (2022) 15:100149. doi: 10.1016/j.eurox.2022.100149, 35493996 PMC9046128

[23] Sjömar J Ottesen H Banik G Rahman AE Thernström Blomqvist Y Rahman SM . Exploring caregivers' experiences of kangaroo mother Care in Bangladesh: a descriptive qualitative study. PLoS One. (2023) 18:e0280254. doi: 10.1371/journal.pone.0280254, 36689433 PMC9870098

[24] Parmar VR Kumar A Kaur R Parmar S Kaur D Basu S . Experience with kangaroo mother care in a neonatal intensive care unit (NICU) in Chandigarh, India. Indian J Pediatr. (2009) 76:25–8. doi: 10.1007/s12098-009-0024-2, 19390999

[25] Moore ER Anderson GC Bergman N Dowswell T. Early skin-to-skin contact for mothers and their healthy newborn infants. Cochrane Database Syst Rev. (2012) 5:CD003519. doi: 10.1002/14651858.CD003519.pub3, 22592691 PMC3979156

[26] Cho ES Kim SJ Kwon MS Cho H. Kim EH Jun EM, et al. The effects of kangaroo Care in the Neonatal Intensive Care Unit on the physiological functions of preterm infants, maternal-infant attachment, and maternal stress. J Pediatr Nurs 2016;31:430–438. doi: 10.1016/j.pedn.2016.02.007 26975461

[27] Sharma D Farahbakhsh N Sharma S Sharma P Sharma A. Role of kangaroo mother care in growth and breast feeding rates in very low birth weight (VLBW) neonates: a systematic review. J Matern Fetal Neonatal Med. (2019) 32:129–42. doi: 10.1080/14767058.2017.1304535, 28274153

[28] Zhu Z Wang X Chen W Pei S Wang Q Guan H . The efficacy of kangaroo-mother care to the clinical outcomes of LBW and premature infants in the first 28 days: a meta-analysis of randomized clinical trials. Front Pediatr. (2023) 11:1067183. Published 2023 Feb 27. doi: 10.3389/fped.2023.1067183, 36923278 PMC10008937

[29] Chan G Bergelson I Smith ER Skotnes T Wall S. Barriers and enablers of kangaroo mother care implementation from a health systems perspective: a systematic review. Health Policy Plan. (2017) 32:1466–75. doi: 10.1093/heapol/czx098, 28973515 PMC5886293

[30] Mhango P Nyondo-Mipando AL. Factors influencing fathers' involvement in the care of hospitalized preterm newborns in Balaka, Malawi. BMC Pediatr. (2023) 23:432. doi: 10.1186/s12887-023-04253-1, 37644490 PMC10463498

[31] Nyondo-Mipando AL Kinshella MW Hiwa T Salimu S Banda M Vidler M . Mothers' quality of life delivering kangaroo mother care at Malawian hospitals: a qualitative study. Health Qual Life Outcomes. (2021) 19:186. doi: 10.1186/s12955-021-01823-8 34321038 PMC8317316

